# Diarrhoeal Disease in Relation to Possible Household Risk Factors in South African Villages

**DOI:** 10.3390/ijerph15081665

**Published:** 2018-08-06

**Authors:** Thandi Kapwata, Angela Mathee, Wouter Jacobus le Roux, Caradee Yael Wright

**Affiliations:** 1 Environment and Health Research Unit, South African Medical Research Council, Johannesburg 2028, South Africa; Thandi.Kapwata@mrc.ac.za (T.K.); Angela.Mathee@mrc.ac.za (A.M.); 2 Environmental Health Department, Faculty of Health Sciences, University of Johannesburg, Johannesburg 2028, South Africa; 3 School of Public Health, University of the Witwatersrand, Johannesburg 2028, South Africa; 4 Natural Resources and the Environment, Council for Scientific and Industrial Research, Private Bag x395, Pretoria 0001, South Africa; wleroux@csir.co.za; 5 Environment and Health Research Unit, South African Medical Research Council, Pretoria 0084, South Africa; 6 Department of Geography, Geoinformatics and Meteorology, Private Bag X20, Hatfield, Pretoria 0028, South Africa

**Keywords:** diarrhoea, water quality, water storage, environmental health, South Africa

## Abstract

Diarrhoeal disease is a significant contributor to child morbidity and mortality, particularly in the developing world. Poor sanitation, a lack of personal hygiene and inadequate water supplies are known risk factors for diarrhoeal disease. Since risk factors may vary by population or setting, we evaluated the prevalence of diarrhoeal disease at the household level using a questionnaire to better understand household-level risk factors for diarrhoea in selected rural areas in South Africa. In a sub-sample of dwellings, we measured the microbial quality of drinking water. One in five households had at least one case of diarrhoea during the previous summer. The most widespread source of drinking water was a stand-pipe (inside yard) (45%) followed by an indoor tap inside the dwelling (29%). Storage of water was common (97%) with around half of households storing water in plastic containers with an opening large enough to fit a hand through. After adjusting for confounders, the occurrence of diarrhoea was statistically significantly associated with sourcing water from an indoor tap (Adjusted Odds Ratio (AOR): 2.73, 95% CI: 2.73, 1.14–6.56) and storing cooked/perishable food in non-refrigerated conditions (AOR: 2.17, 95% CI: 2.17, 1.44–3.26). The highest total coliform counts were found in water samples from kitchen containers followed by stand-pipes. *Escherichia coli* were most often detected in samples from stand-pipes and kitchen containers. One in four households were at risk of exposure to contaminated drinking water, increasing the susceptibility of the study participants to episodes of diarrhoea. It is imperative that water quality meets guideline values and routine monitoring of quality of drinking water is done to minimise diarrhoea risk in relevant rural communities. The security of water supply in rural areas should be addressed as a matter of public health urgency to avoid the need for water storage.

## 1. Introduction

Water-related diseases have a significant impact on human health globally. It is estimated that 1.7 billion children suffer from diarrhoeal disease annually [1,2]. Diarrhoeal disease is of particular concern in developing countries [3] as a result of several factors, including poverty, poor sanitation, lack of hygiene, inadequate availability of water and non-existent or unreliable supply of piped water [4,5]. Nearly three-quarters of the global childhood diarrhoea mortality burden, described in the 2015 Global Burden of Disease Study, occurred in south-east Asia and Africa [6]. In South Africa, diarrhoeal disease is the third leading cause of death among children under five years of age [7]. It is also the eighth most frequent cause of death in the country, accounting for 3% of total deaths among individuals of all ages [8].

Previous studies [9,10] have found several risk factors for diarrhoea, including low economic status [9], a lack of education, poor water storage practices, not treating water in the home, overcrowding and a high number of children under five years of age living in a household [9]. Other studies have also assessed risk factors for diarrhoea by type of pathogen where rotavirus and *Escherichia coli* (*E. coli*) were the two most common pathogens associated with moderate-to-severe diarrhoea in low-income countries [1]. Lanata et al. [11] found that *E. coli* caused more than half of all diarrhoeal deaths in children under five years and had also been associated with increased risk of diarrhoeal mortality in infants aged 0 to 11 months [12]. Other biological disease agents that contaminate drinking water, such as total coliforms and somatic coliphages, also increase the risk of diarrhoeal disease [13].

Since risk factors vary based on the setting and characteristics of the target population [14], we evaluated the prevalence of diarrhoeal disease at household level in villages in Limpopo province in South Africa, to better understand the determinants of diarrhoea in that part of the country. In a sub-sample of households, we also investigated the microbial quality of drinking water by quantifying the presence of selected bacterial indicators. Furthermore, we looked at the impact of the location of drinking water access points on microbial quality for the first time in this setting. We identify possible actions and/or routes for intervention to reduce the prevalence of diarrhoea at the community level in the study setting.

## 2. Materials and Methods

### 2.1. Ethical Statement

Research ethics clearance for the study was granted by the South African Medical Research Council Ethics Committee (Certificate number: EC005-3/2014).

### 2.2. Study Area

**Figure 1:**
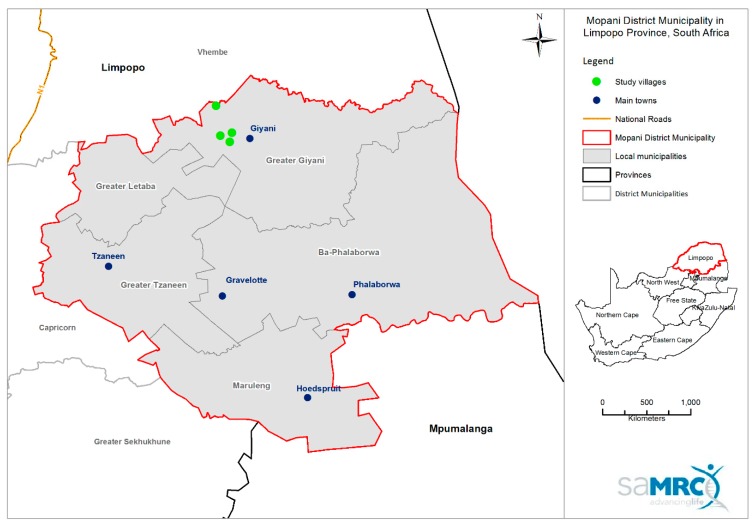
Map showing the study sites (green dots) in relation to Giyani town in Limpopo province, South Africa.

Households were located in four villages around a town called Giyani located in the Mopani District Municipality of Limpopo province (Figure 1).

### 2.3. Data Collection

A cluster sampling method was used to select 400 households from four villages (100 households per village) around Giyani town. Following written informed consent, a self-report questionnaire was administered in April (autumn) 2017 to the primary caregiver or head of household (individuals over 18 years of age), to obtain information on the households socio-demographic and socio-economic status, the health status of household members and risk factors identified from the literature for diarrhoeal disease. The definition of a household was ‘a group of people eating meals together’.

The self-report questionnaire was adapted from two existing questionnaires [15,16] and was conducted in a face-to-face interview (between trained fieldworker and primary household caregiver). We extracted data pertaining to demographics, water collection, storage, use and household diarrhoea prevalence (see Table 1 and Table 2) from the full questionnaire (including captured additional environmental risk factors such as air pollution; full questionnaire provided in Supplementary Materials). The term ‘diarrhoea’ was not specifically defined in the questionnaire, nor was the case of self-reported diarrhoea validated. The question asked which individuals in the household had experienced ‘diarrhoea’ in the past summer and these data were re-categorised to binary, such that, if anyone in the household had experienced diarrhoea in the past summer then the response was ‘yes’ (and vice versa). This was done in part because the individual prevalence of diarrhoea was very low, and also because the primary sampling unit was the household and not the individual.

### 2.4. Water Sample Collection

In a separate field campaign (carried out in February/March 2017) a water sample was taken from 200 (random selection of 50 per village) households’ usual source of water for drinking and cooking. The location of this water source was recorded by the fieldworker. Sources of water included a tap inside the dwelling (defined as an indoor tap), a container in the kitchen, a tap inside the yard where the household is located (defined as stand-pipe (in yard)), a communal tap that serves several households (defined as a stand-pipe (communal)), or an outdoor water storage tank which stores harvested rainwater (locally known as a ‘JoJo’ tank).

### 2.5. Microbiological Analysis of Water Samples

In line with the United States Environmental Protection Agency methodology [17], *E. coli* and total coliforms were quantified using the Colilert™ (IDEXX Laboratories Inc., Westbrook, ME, USA) Most Probable Number (MPN) method (IDEXX Laboratories, Westbrook, ME, USA) using 18 sachets and the Quanti-Tray™ (IDEXX Laboratories Inc., Westbrook Inc., ME, USA) 2000 system. Results were read within 18 to 24 h as the most probable number (un-altered) except for counts of <1/100 mL which were interpreted as 0/100 mL. Somatic coliphages were determined using a double agar layer plaque assay with *E. coli* (ATCC 15597) employed as host bacterium. Constituents used to make up the growth media included agar bacteriological, sodium chloride and glucose (Merck, Darmstadt, Germany) and tryptone (Oxoid, Basingstoke, UK). Plates were incubated for 18 to 20 h at 35 °C, then clear zones (plaques) were counted and expressed as plaque-forming units per volume of inoculated water.

### 2.6. Data Management and Statistical Analyses

Data management and statistical analyses were done using Stata 14.0 [18]. Descriptive and regression analysis were computed using complex survey (‘svy’ command) data analysis to account for cluster design [19,20,21]. Sampling weight, used to weight the sample back to the population from which the sample was drawn, and primary sampling unit, the first unit that is sampled in the design, were two design parameters specified in ‘svy’. While differences between villages were controlled for, analyses by village were not possible due to low numbers by village sample and question category.

The primary outcome variable was whether or not an incident of diarrhoeal disease had affected any member of the household during the summer preceding the questionnaire interview. Associations between diarrhoea and several risk factors were evaluated by conditional logistic regression, and a multiple logistic regression model was fitted using a stepwise backward procedure. Model building proceeded backwards from a first “full” model including all of the variables found to have a *p*-value below 0.20 in the univariate analysis, and from which the non-statistically significant variables were then removed sequentially. A *p*-value below 0.05 was considered statistically significant in the multivariate analysis. We adjusted for potential confounders that could play a role in the occurrence of diarrhoea through an indirect link. These included socio-economic status, i.e., household income and dependence on state assistance in the form of grants, household size and the number of children under five years of age living in a household [9,22,23,24]. The goodness of fit of final multivariate logistic models was assessed using the command “estat gof” in Stata [18], an adaptation to the Hosmer and Lemeshow’s goodness of fit test which is suitable for survey data analysis. All water samples were analysed for total coliforms, *E. coli* and somatic coliphages. Somatic coliphages were not detected in any of the water samples.

## 3. Results

### 3.1. Sample Description

Self-reported prevalence of diarrhoea at household level was 20% (*n* = 82, total *n* = 408) (Table 1). About half of the households comprised five or more occupants. Nearly one-third of households had no source of income, and a further 24% earned ZAR1000 (~USD75) or less per month. The majority (70%) of households received a state-sponsored child grant.

### 3.2. Household Water Access and Storage

The most widely prevalent source of drinking water was a standpipe (in yard) followed by an indoor tap (Table 2). Storage of water for cooking and/or drinking purposes collected from indoor taps or stand-pipes was common. Around half of the households stored water in plastic containers with openings large enough to pass a hand through. Univariate regression showed that source of drinking water, water storage practices and treatment, for example with bleach, chlorine etc., and cooked/perishable food storage practices were statistically significant risk factors for diarrhoea (Table 2).

Table 3 presents multivariate regression results before and after adjusting for confounders. Diarrhoea was statistically significantly associated with sourcing water from an indoor tap (AOR: 2.73, 95% CI: 2.73, 1.14–6.56) and storing cooked/perishable food in a non-refrigerated cupboard (AOR: 2.17, 95% CI: 2.17, 1.44–3.26). The goodness of fit test for the model produced a *p*-value > 0.05.

### 3.3. Assessment of Drinking Water Quality in A Sub-Sample of Households

In total, 192 water samples were collected. The highest microbial risk was found in water samples from kitchen containers followed by samples from standpipes. Total coliform counts exceeded 100 counts/100 mL water in 21% and 17% of these samples, respectively. Microbial results were compared to the South African Water Quality Guidelines (SAWQG) for domestic water [25] and categorised according to known associated health risks based on the guidelines. For total coliforms, 0–5 counts/100 mL is associated with negligible risk of microbial infection. A total of 6–100 counts/100 mL is indicative of inadequate treatment, post-treatment contamination or growth in the distribution system with the risk of infectious disease transmission present with continuous exposure and a slight risk present with occasional exposure [25]. Similarly, samples with total coliform counts exceeding 100/100 mL are indicative of a significant and increasing risk of infectious disease transmission [25]. Effects on human health associated with *E. coli* were categorised according to 0 counts/100 mL with negligible risk of microbial infection and 1–10 counts/100 mL indicative of a slight risk of microbial infection with continuous exposure and negligible effects with occasional or short-term exposure [25]. Samples with *E. coli* counts between 11 and 20 counts/100 mL were considered to have some risk of infectious transmission with continuous exposure and slight risk with occasional exposure; and samples with more than 20 counts/100 mL were indicative of significant and increasing risk of infectious disease transmission such that as faecal coliform levels increase, the required amount of water ingested to cause infection decreases [25].

*Escherichia coli* were most often detected in water samples taken from stand-pipes and kitchen containers (Table 4). In these samples, *E. coli* was present at counts that exceeded the target water quality range of <1/100 mL [26,27,28]. Although *E. coli* is often used as an indicator of faecal pollution, and therefore also an indicator of water-borne pathogens, some strains can also cause intestinal and extra-intestinal disease [25]. Our results showed that ~50 households who participated in this study were at risk of such diseases due to exposure to contaminated drinking water.

## 4. Discussion

This study assessed diarrhoea prevalence at the household level and identified household risk factors associated with diarrhoea. We further assessed microbial water quality in a convenient sub-sample of households. The majority of households had a low socio-economic status, with nearly one-third of respondents reporting that their households had no source of income and no form of schooling.

The prevalence of caregiver-reported diarrhoea in summer in households was 20%. Diarrhoea was significantly associated with households reportedly obtaining water from indoor taps. This could have been because households assumed that water from the tap was safe and therefore tended not to boil or treat the water [1]. Intermittent water supply is a common problem in rural areas in Limpopo [29]. Frequent interruptions in water supply also affects the quality of piped water due to the intrusion of contaminants into the distribution network during times of low pressure or when the water supply is turned off [30,31]. Illegal connections to water pipes, common in parts of South Africa [32], also affects the continuity of the water supply. Biofilm regrowth in pipes when the water supply is turned off further contributes to poor water quality [33]. Poorly maintained and aging infrastructure may result in leaks which lead to contamination of piped water [29,33]. These findings were substantiated with microbial results showing that a large number of water samples from stand-pipes linked to the water reticulation system had detectable levels of either total coliforms, *E. coli* or both. Nearly one in three households of the sub-sample of households exceeded the target range of 0–5 counts of total coliform/100 mL of water. *Escherichia coli* should not be detectable in drinking water, yet 28% of households had water that exceeded this target. This is not in compliance with the SAWQGs [25] and indicates that water from piped distribution systems had low microbial water quality. A similar study [34] in a low socio-economic setting in the Free State province also found that the microbial quality of piped water was poor. There, the total coliform count in more than 50% of samples from the municipal water supply was found to contain more than 5 coliforms per 100 mL of water.

Storing water for cooking and drinking purposes was practised by the majority of households, including those living in dwellings supplied with an indoor tap. Water storage creates additional contamination pathways and leads to an increase in associated health risks. Microbial analyses indicated that water samples from storage containers had high counts of total coliforms. This could be due to poor water handling practices, as well as obtaining the water from the points of supply observed to be contaminated, identified by our study findings. A similar study in rural Limpopo [34] also found that there was an increase in indicator micro-organism counts in water storage containers compared to the initial source (in that case it was indoor tap water). Poor microbial quality of stored water was also identified in Cambodia [35] where researchers found *E. coli* counts in samples obtained from piped water were lower than counts in stored piped water (*p* < 0.0005). Furthermore, not treating stored drinking water has been significantly associated with the prevalence of diarrhoea among children [36]. Studies have shown that storing water leads to a deterioration of water quality because of recontamination in the home [37,38,39]. Factors that could increase contamination of stored water include the method used to obtain water from the container [34], size of the storage vessel mouth [38], higher temperatures leading to bacterial regrowth within the container [40], increased storage times and inadequate hand washing [41]. Half of the households in our study used plastic storage containers with an opening wide enough to fit a hand through, leading to an elevated risk of contamination of water stored in an uncovered, wide-necked container. Similarly in an earlier Limpopo study [41,42] significantly higher levels (almost three times higher) of total coliform bacteria were found in water stored in wide-necked, compared to narrow-necked containers. Although storing water is not ideal, often the intermittent supply of water in rural communities necessitates water storage.

A second risk factor significantly associated with diarrhoea was the practice of storing cooked or perishable food in unrefrigerated conditions. Among rural households in Malawi, high numbers of pathogens were found in cooked food stored at room temperature [43]. Among our respondents who selected ‘other’ as a means of storing cooked or perishable food, storage options may have been a deep freeze, or in pots, plastic bowls, jugs and buckets on top of tables and countertops. It may also have been that the choice of ‘other’ was made by respondents who do not store food. Poor food hygiene practices are regarded as major contributors to diarrhoea [44], and households should be encouraged to keep cooked or perishable food in a refrigerator or cool conditions where possible.

Our study findings were constrained by several limitations. While we did attempt to link water quality results to the households’ questionnaire data (i.e., caregiver-reported diarrhoeal disease and household risk factors); it was not possible to confirm the source of the water sample to where the household typically collected their water. In most instances, the household member spoke about where the household typically collected/obtained their water. Water was sampled from the household’s main water source, which was most often the water storage container in the kitchen. In the instance that we could match the household diarrhoeal disease cases to water microbial data, there were only 37 cases making further analysis impractical. We only conducted water testing on a sub-sample of households due to financial constraints (additional testing of water from indoor taps is important), and we did not verify household cases of diarrhoea reported by the caregivers with clinic or hospital data due to the complexity of identifying the appropriate healthcare facility.

Our results highlight a quadruple risk in relation to water quality. First, there was evidence of poor water quality at source (tap), and secondly, there was declining water quality from storage and handling practices. There were water quality risks from large-diameter openings of water storage containers which permits contamination from hands and utensils used for drinking/scooping water. This also leads to the intrusion of micro-organisms and insects when lids were removed. Lastly, there was an additional risk from frequent supply interruptions leading to lowered water availability (increasing the need for water storage) which is also associated with increased risk of skin, eye and other infections [45,46]. Given our study findings, it is imperative that water supply to households in rural settings complies with SAWQGs. Secondly, the security of that water supply should be addressed as a matter of public health urgency to avoid the need for water storage. Finally, there is a need to provide informed advice on the storage and treatment of water among households in rural settings.

## 5. Conclusions

This study characterized risk factors for diarrhoea pertinent to a rural South African setting. Risk factors that remained significant after adjusting for confounders were sourcing water from an indoor tap and storing cooked/perishable food in non-refrigerated cupboards. Microbial water quality of water samples from two water sources, namely water stored in containers in kitchens and stand-pipes, was poor with the microbial quality not meeting the national water guideline values recommended for total coliforms and *E. coli*, making affected rural residents vulnerable to episodes of diarrhoea. It is imperative that the water supply to households in rural settings comply with water quality guideline values and that routine monitoring of the quality of drinking water by testing for the presence of organisms and other physical contents is undertaken. The security of the water supply in rural areas should be addressed as a matter of public health urgency to avoid the need for water storage. There is also a need to provide informed advice on storage and treatment of water among households in rural settings when storage is unavoidable.

## Figures and Tables

**Table 1:** Prevalence of primary caregiver-reported diarrhoea and demographic characteristics of households (*n* = 408).

Characteristics	Number of Households	
	*n*	%
Prevalence of self-reported diarrhoea (for any individual in the household during the past summer)	82	20
Gender of respondent:		
Male	79	19
Female	329	81
Age of respondent in years:		
18–24 years	45	11
25–29 years	46	11
30–39 years	71	17
40–49 years	49	12
50–59 years	80	20
≥60 years	117	29
Number of people in household:		
≤5 people ^!^	194	47
6–7 people	167	41
8–10 people	42	10
>10 people	5	1
Number of children <5 years of age in household:		
0 children ^#^	239	58
2–3 children	167	41
>3 children	2	0.4
Number of years occupying dwelling:		
≤10 years	69	17
11–20 years	80	20
>20 years	259	63
Level of education of respondent:		
No schooling	123	30
Primary school	53	13
High school	194	48
Tertiary education	29	7
Average monthly income of household (excluding grants and pensions):		
No income	121	30
ZAR1000 or less	98	24
ZAR1001–ZAR5000	100	25
≥ZAR5001	17	4
Do not know	72	18
Proportion of households with a member receiving the following grants:		
Old age pension	179	44
Disability	17	3
Child support	284	70
Other	11	3

Notes. ^#^ No household had one child in the home hence this category is not reported; ^!^ this category of ≤5 people cannot be broken down further due to question design.

**Table 2:** Results of univariate logistic regression for household risk factors in relation to household diarrhoea prevalence.

Question and Responses	Prevalence	Univariate Results		
	*n* (%)	OR	95% CI	*p*-Value
Where do you mainly get your drinking water from:				
Stand-pipe (in yard) ^&^	184 (45)	1	-	-
Indoor tap	122 (30)	2.66	2.05–3.45	<0.001
Stand-pipe (communal)	75 (19)	1.57	0.90–2.72	0.080
Private water seller	12 (3)	1.75	0.16–18.70	0.510
Borehole	11 (3)	1.38	1.05–1.81	0.030
Water tanker/truck *	2 (0.5)	*	*	*
Do you store water for drinking or cooking in a container:				
Yes ^&^	394 (97)	1	-	-
No	14 (3)	1.20	0.03–43.22	0.880
If you store water indoors, does the container have a lid:				
Yes	369 (94)	1	-	-
No	25 (6)	1.07	0.61–1.87	0.740
If you store water indoors, is the water container:				
Large hole-big enough to get hand in	266 (68)	1	-	-
Small hole-too small to get hand in	128 (33)	1.91	1.00–3.63	0.050
When do you add a fresh supply of water to the container:				
If totally empty	339 (86)	1	-	-
If partially empty	55 (14)	1.39	0.22–0.28	<0.001
How often do you wash your drinking water storage container:				
Daily	26 (7)	0.72	0.38–1.35	0.200
Once or twice a week	237 (60)	-	-	-
Monthly	91 (23)	0.99	0.32–3.07	0.970
Never	39 (10)	1.09	0.35–3.36	0.820
Do you boil stored water before drinking it:				
No ^&^	373 (95)	1	-	-
Yes	21 (5)	0.69	0.27–1.72	0.280
Do you add bleach to stored water before drinking it:				
No ^&^	341 (87)	1	-	-
Yes	53 (13)	1.83	0.86–3.90	0.080
If you store food, do you store it in a sealed container:				
No	20 (5)	1.17	0.32–4.29	0.730
Yes ^&^	388 (95)	-	-	-
When you need to store cooked/perishable food, do you store the food in a:				
Fridge	353 (87)	1	-	-
Food cupboard	42 (10)	1.58	0.90–2.78	0.080
Other (No further details provided)	13 (3)	1.8	0.63–5.17	0.170
What type of toilet does the household mainly use:				
Flush (Not specified indoor or outdoor)	12 (3)	4.60	1.80–11.73	0.014
Pit latrine	391 (96)	-	-	-
Communal toilet *	3 (0.7)	*	*	*
Open field *	2 (0.4)	*	*	*
How often do you clean your toilet:				
Weekly	274 (67)	1	-	-
Daily	75 (18)	1.02	0.72–1.44	0.890
Seldom	59 (15)	0.67	0.56–0.81	0.010

Notes. OR is Odds Ratio and CI is Confidence Interval; the reference category is indicated by OR = 1; * indicates categories that were excluded from univariate regression due to low (<10) number of observations but are shown here for completeness. Risk factors with *p* > 0.20 were included in multivariate regression; ^&^ this category was the normative category for the sampled households and had the largest number of responses; therefore, it was selected as the reference category.

**Table 3:** Results of multivariate logistic regression for household risk factors in relation to household diarrhoea prevalence.

Variable	OR ^^^	95% CI ^@^	*p*-Value	AOR ^&^	95% CI	*p*-Value
Do you mainly get your drinking water:						
From an indoor tap	2.51	1.51–4.15	0.010	2.75	1.13–6.73	0.040
From a stand-pipe (communal)	1.40	0.59–3.35	0.341	0.92	0.37–2.30	0.800
From a borehole	1.34	0.71–2.52	0.237	0.67	0.28–1.61	0.240
Is the water container:						
Small hole, too small to get hand through	1.94	0.96–3.93	0.058	1.29	0.69–2.36	0.280
When do you add a fresh supply of water to the container:						
If partially empty	1.75	0.85–3.61	0.090	1.61	0.46–5.62	0.309
How often do you wash your drinking water storage container:						
Once or twice a week	1.01	0.49–2.06	0.981	0.71	0.35–1.45	0.220
What do you do with water stored in a container before drinking it:						
Add ‘Jik’ (bleach)	2.13	0.67–6.82	0.130	2.41	0.53–11.00	0.160
Do you store cooked/perishable food in a food cupboard (i.e., non-refrigerated conditions):						
Yes	2.04	1.02–4.06	0.046	2.14	1.44–3.19	0.009
Do you store the cooked/perishable food in another way:						
Yes	1.91	0.40–9.08	0.275	1.99	0.34–11.43	0.30
What type of toilet does the household mainly use:						
Flush	0.30	0.02–4.05	0.230	0.24	0.007–7.48	0.28
How often do you clean your toilet:						
Seldom	0.83	0.63–1.11	0.136	0.78	0.18–3.36	0.63

Notes. ^^^ OR is Odds Ratio and ^@^ CI is the Confidence Interval; ^&^ AOR is Adjusted Odds Ratio where we adjusted for household income and dependence on state assistance in the form of grants, family size and the number of children under five years of age living in a household.

**Table 4:** Results of the tests for presence of total coliforms and *E. coli* in water sampled from accessible water points in a sub-sample (*n* = 192) of water points used by households.

Point of Tested Water Sample	Number of Samples by Total Coliforms Range (Counts/100 mL)			Number of Samples by *E. coli* Range (Counts/100 mL)			
	0–5	5–100	>100	0	1–10	11–20	>20
	*n* (%)	*n* (%)	*n* (%)	*n* (%)	*n* (%)	*n* (%)	*n* (%)
Indoor tap (*n* = 3) ^!^	2 (67)	0 (0)	1 (33)	2 (67)	0 (0)	0 (0)	1 (33)
Stand-pipe (inside yard) (*n* = 98)	57 (58)	13 (13)	28 (28)	70 (71)	16 (16)	1 (1)	11 (11)
Stand-pipe (communal) (*n* = 8)	2 (25)	3 (38)	3 (28)	7 (88)	0 (0)	0 (0)	1 (13)
Outdoor water tank (*n* = 2)	1 (50)	1 (50)	0 (0)	2 (100)	0 (0)	0 (0)	0 (0)
Kitchen container (*n* = 57)	7 (12)	14 (25)	36 (63)	34 (60)	10 (18)	2 (4)	11(19)

Notes: ^!^ These *n* values do not add up to 192 because not all samples had a source captured.

## Supplementary Materials

The following are available online at http://www.mdpi.com/1660-4601/15/8/1665/s1, Figure S1: Full study questionnnaire.
