# Supplementary material for: Diarrhoeal Disease in Relation to Possible Household Risk Factors in South African Villages
Source: Int J Environ Res Public Health. 2018 Aug 6;15(8):1665. doi: 10.3390/ijerph15081665 (PMC6121382; doi:10.3390/ijerph15081665)
Supplement: Supplementary file 1 [file ijerph-15-01665-s001.pdf]

Check out the Mobenzi Help Centre
For tutorials, guides and examples on how to get the most out of Mobenzi Researcher, spend a few minutes reviewing our Help Centre . We're continually adding new material and it's the fastest, easiest way to get assistance when you need it.
CLOSE NOTICE

IDEWS 2017

Last Modified by: Hendrik Snyman **on** 23 Mar 2017 11:22:00 **Revision number:** 473 **Field Count:** 141

Section 1. Household identification and selection

1.1 Place

In which village are you?

Expects a single option response (**required**)

- ☐ 1 [1]
- ☐ 2 [2]
- ☐ 3 [3]
- ☐ 4 [4]

1.2 study_id_number

What is the study ID number of the household where you are?

Expects a numeric response (**required**)

1.3 gps_location

Please capture the GPS location of the household:

Expects a latitude and longitude coordinate (optional)

1.4 respondent_qualification_wiling

Is there someone that's willing to answer the questions? We prefer to speak to the person in the household who makes decisions about energy usage or who participates in cooking and heating. Or if this is not possible to any other informed adult.

Expects a single option response (**required**)

- ☐ Yes [1]
- ☐ There's nobody home [2]
- ☐ No household member wants to take part [3]
- ☐ There is no suitable respondent [4]
- ☐ I feel unsafe to enter this house [5]

Branches

If response **Not Equal 'Yes [1]'** then skip to ***end (15.1)***

1.5 respondent_name_surname

What is the respondent's name and surname?

Expects a single line text response (**required**)

Section 2. Demography and socio-economic status

2.1 country_born

In which country were you born?

Expects a single option response (**required**)

☐ South Africa [1]

☐ Other [2]

Prerequisites
Skip when ***country_born* (2.1)** **Not Equal 'Other [2]'**

2.2 country_other

Please specify in which country you were born

Expects a single line text response (**required**)

Prerequisites
Skip when ***country_born* (2.1)** **Not Equal 'South Africa [1]'**

2.3 country_province

In which PROVINCE were you born?

Expects a single option response (**required**)

☐ Eastern Cape [1]

☐ Free State [2]

☐ Gauteng [3]

☐ KwaZulu-Natal [4]

☐ Limpopo [5]

☐ Mpumalanga [6]

☐ Northern Cape [7]

☐ North West [8]

☐ Western Cape [9]

☐ Not born in South Africa [10]

2.4 number_people

How many people live on this stand, i.e. within the boundaries of the yard?

Expects a numeric response (**required**)

2.5 number_households

How many separate households live on this plot? A household is a group of people who usually eat together.

Expects a numeric response (**required**)

2.6 households_family

Please complete the sentence: Are all households

Expects a single option response (**required**)

☐ Part of the same family [1]

☐ not family members – simply renting space [2]

2.7 main_household

The remaining questions of this section is about the main household.

2.8 people_main

How many people, including you, make up this main household?

Expects a numeric response (**required**)

2.9 household_children

How many children under five years of age are part of this household? If there are no children under five, type 0

Expects a numeric response (**required**)

2.10 household_language

What is the main language spoken in this (the main/primary) household?

Expects a single option response (**required**)

- ☐ Afrikaans [1]
- ☐ English [2]
- ☐ Ndebele [3]
- ☐ Sepedi [4]
- ☐ Sesotho [5]
- ☐ Setswana [6]
- ☐ Swati [7]
- ☐ Tsonga [8]
- ☐ Venda [9]
- ☐ Xhosa [10]
- ☐ Zulu [11]
- ☐ Other [12]

2.11 household_years

How long has this household been living in this house?

(**required**)

2.12 instruction_people

The following section will repeat for the number of persons in this household.

2.13 instruction_write down persons

Please write down the names of every person who lives here, starting with the respondents as Person 1, and there after from the oldest to youngest member.

Repeat this section for value of ***people_main (2.8)***

Section 3. Household member information

3.1 person_name

What is REPEAT IDX name?

Expects a single line text response (**required**)

3.2 person_age

How old is REPEAT IDX ?

Expects a numeric response (**required**)

3.3 person_sex

Is REPEAT IDX male or female?

Expects a single option response (**required**)

- ☐ Male [1]
- ☐ Female [2]

3.4 person_education

What is the highest achieved educational qualification for REPEAT IDX ?

Expects a single option response (**required**)

- ☐ None [1]
- ☐ Primary [2]
- ☐ Secondary [3]
- ☐ Tertiary [4]
- ☐ Not applicable [5]
- ☐ Child is under 5 years old [6]

3.5 person_activity

Please state the main weekly activity for REPEAT IDX ?

Expects multiple selected options (**required**)

- ☐ Full time job [1]
- ☐ Part time job [2]
- ☐ Unemployed [3]
- ☐ Housewife / husband [4]
- ☐ Informal job [5]
- ☐ Tertiary education [6]
- ☐ School [7]
- ☐ Crèche [8]
- ☐ Other [9]

Section 4. Household information

4.1 household_income

What is the average monthly income for this household - excluding grants & pensions?

Expects a single option response (**required**)

- ☐ No income [1]
- ☐ < R1000 [2]
- ☐ R1001- R5000 [3]
- ☐ > R5001 [4]
- ☐ Don't know [5]

4.2 household_Oldage_grant

How many people in the household receive Old age grant/pension?

Expects a numeric response (**required**)

4.3 household_grand_Disability

How many people in this household receive disability grant?

Expects a numeric response (**required**)

4.4 household_grant_child

How many people in this household receive Child support grant?

Expects a numeric response (**required**)

4.5 household_grant_other

Does anyone in this household reveive other grants?

Expects a single option response (**required**)

- ☐ Yes [1]
- ☐ No [2]

Prerequisites
Skip when ***household_grant_other* (4.5) Not Equal 'Yes [1]'**

4.6 Household_grant_specify

Please specify what grant

Expects a single line text response (**required**)

4.7 household_own

Please state whether members of this household OWN any of the following items (in working order)

Expects multiple selected options (**required**)

- ☐ Radio [1]
- ☐ Television [2]
- ☐ Satellite television (e.g. DSTV or M-Net) [3]
- ☐ Refrigerator [4]
- ☐ Washing machine [5]
- ☐ Video/DVD machine [6]
- ☐ Microwave [7]
- ☐ Car [8]
- ☐ Computer [9]
- ☐ Telephone (landline) [10]
- ☐ Cellular phone (plain) [11]
- ☐ Cellular smart phone [12]
- ☐ iPad or Tablet [13]
- ☐ Internet access [14]

Section 5. Environmental services and practices

5.1 water_drinking

Where do you mainly get your drinking water from?

Expects a single option response (**required**)

- ☐ Tap inside dwelling [1]
- ☐ Outdoor on-site tap [2]
- ☐ Communal tap-offsite [3]
- ☐ Private water seller [4]
- ☐ Water tanker/ truck [5]
- ☐ Borehole [6]
- ☐ Rain water tank [7]
- ☐ River/stream/dam [8]
- ☐ Other [9]

5.2 water_available

If you main source of drinking water is not available, where do you get your drinking water from?

Expects a single option response (**required**)

- ☐ Tap inside dwelling [1]
- ☐ Outdoor on-site tap [2]
- ☐ Communal tap-offsite [3]
- ☐ Private water seller [4]
- ☐ Water tanker/ truck [5]
- ☐ Borehole [6]
- ☐ Rain water tank [7]
- ☐ River/stream/dam [8]
- ☐ Other [9]

5.3 water_storage

Do you store water for drinking or cooking in a container? (such as a bucket or 5-litre water container)

Expects a single option response (**required**)

- ☐ Yes [1]
- ☐ No [2]

Branches

If response **Not Equal 'Yes [1]'** then skip to ***water_swim (5.14)***

5.4 water_container

Is the container

Expects a single option response (**required**)

☐ Metal, totally open [1]

☐ Metal, large hole-Big enough to get hand in [2]

☐ Metal, small hole-Too small to get fist in [3]

☐ Plastic, totally open [4]

☐ Plastic, large hole-Big enough to get hand in [5]

☐ Plastic, small hole-Too small to get fist in [6]

☐ Other, totally open [7]

☐ Other, large hole-Big enough to get hand in [8]

☐ Other, small hole-Too small to get fist in [9]

☐ Not applicable [10]

5.5 Container_lid

Does the container have a lid?

Expects a single option response (**required**)

☐ Yes [1]

☐ No [2]

5.6 container_clean

What do you do or add to the water to make it and keep it clean before drinking

Expects multiple selected options (**required**)

☐ Nothing [1]

☐ Boil the water [2]

☐ Add Jik [3]

☐ Add Chlorine [4]

☐ Add Jeyes Fluid [5]

☐ Other [6]

Prerequisites
Skip when *container_clean (5.6)* **Excludes 'Other [6]'**

5.7 container_clean_specify

What else do you do or add to the water to make it and keep it clean?

Expects a single line text response (**required**)

5.8 container_wash

How often do you wash your drinking water storage container?

Expects a single option response (**required**)

☐ Daily [1]

☐ Once or twice a week [2]

☐ Monthly [3]

☐ Never [4]

☐ Other [5]

Prerequisites
Skip when *container_wash (5.8)* Not Equal 'Other [5]'

5.9 container_wash_other

You chose other, please explain when you wash your container

Expects a single line text response (**required**)

5.10 container_fresh

When do you add a fresh supply of water to the container?

Expects a single option response (**required**)

☐ If totally empty [1]

☐ When half empty [2]

5.11 container_take_water

How do you take water out of the container?

Expects a single option response (**required**)

☐ Using a utensil (scoop, cup, jug) [1]

☐ Hands [2]

☐ Other [3]

Prerequisites
Skip when *container_take_water (5.11)* Not Equal 'Other [3]'

5.12 container_take_water_other

You chose other, pleae specify how you take water out of the container

Expects a single line text response (**required**)

5.13 container_cup

Is there one special cup that everyone uses for scooping and drinking water from the container?

Expects a single option response (**required**)

☐ Yes [1]

☐ No [2]

5.14 water_swim

Does anyone in the family ever swim in a nearby water body such as a water reservoir, dam, river?

Expects a single option response (**required**)

☐ Yes [1]

☐ No [2]

5.15 household_toilet

What type of toilet does the household mainly use?

Expects a single option response (**required**)

- ☐ Flush [1]
- ☐ Chemical [2]
- ☐ Pit latrine [3]
- ☐ Communal toilet [4]
- ☐ Bucket [5]
- ☐ Open field [6]
- ☐ Other [7]

5.16 refuse_located

Where is your refuse container (bin) located?

Expects a single option response (**required**)

- ☐ Kept indoors [1]
- ☐ Outdoors [2]
- ☐ Do not own a bin [3]

Branches

If response **Equals 'Do not own a bin [3]'** then skip to ***refuse_collection (5.18)***

5.17 refuse_lid

Does your bin have a properly fitting lid?

Expects a single option response (**required**)

- ☐ Yes [1]
- ☐ No [2]

5.18 refuse_collection

How often is your waste/refuse collected by local authority?

Expects a single option response (**required**)

- ☐ Daily [1]
- ☐ Weekly [2]
- ☐ Monthly [3]
- ☐ Sporadically [4]
- ☐ Never [5]
- ☐ Other [6]

Prerequisites

Skip when ***refuse_collection (5.18)*** **Not Equal 'Other [6]'**

5.19 refuse_collection_other

You chose other, please specify when

Expects a single line text response (**required**)

5.20 refuse_not_collected

If your waste is not collected, how do you dispose of your waste?

Expects multiple selected options (**required**)

- ☐ Burn [1]
- ☐ Bury [2]
- ☐ Dump (close to the house) [3]
- ☐ Dump within 100m of house [4]
- ☐ Dump at municipal dump site [5]
- ☐ Dump in river [6]
- ☐ Other [7]

Prerequisites
Skip when *refuse_not_collected (5.20)* Excludes 'Other [7]'

5.21 refuse_not_collected_other

You use another option, please specify

Expects a single line text response (**required**)

5.22 animals_yard

What types of pets/ animals are in the house or the yard?

Expects multiple selected options (**required**)

- ☐ Cats [1]
- ☐ Dogs [2]
- ☐ Rodents e.g. Hamster, mice [3]
- ☐ Goats [4]
- ☐ Sheep [5]
- ☐ Chickens [6]
- ☐ Birds (such as budgies/pigeons) [7]
- ☐ None [8]
- ☐ Other [9]

Prerequisites
Skip when *animals_yard (5.22)* Excludes 'Other [9]'

5.23 animals_yard_other

You chose other animals, please specify

Expects a single line text response (**required**)

Section 6. Housing infrastructure

6.1 instruction_fieldworker

Please record the type of dwelling used by the main household. (please choose one)

Expects a single option response (**required**)

- ☐ Formal house built by a professional builder [1]
- ☐ Formal house that was self-built [2]
- ☐ Informal dwelling [3]
- ☐ Backyard dwelling-formal [4]
- ☐ Backyard dwelling-informal [5]
- ☐ Flat [6]
- ☐ Traditional dwelling [7]
- ☐ Other [8]

Prerequisites
Skip when ***instruction_fieldworker (6.1)*** **Not Equal 'Other [8]'**

6.2 instruction_specify

What type of dwelling is used?

Expects a single line text response (**required**)

6.3 dwelling_age

Approximately how old is the house in years?

Expects a numeric response (**required**)

6.4 dweilling_rooms

How many separate rooms are there in this dwelling/ house?

Expects multiple selected options (**required**)

- ☐ Kitchen (for cooking only) [1]
- ☐ Bathrooms / toilets [2]
- ☐ Dining rooms [3]
- ☐ Lounge [4]
- ☐ Bedrooms [5]

6.5 dwelling_mould

Is there mould or mildew (usually black or brown spots) growing on any damp or moist surface inside the home? (e.g. on walls, wallpaper, carpets, ceilings, shower, curtains, etc.)

Expects a single option response (**required**)

- ☐ Yes [1]
- ☐ No [2]

6.6 dwelling_dampness

Do you have any dampness in the dwelling?

Expects a single option response (**required**)

- ☐ Yes [1]
- ☐ No [2]

6.7 dwelling_leaks

Do you have any leaks in the roof or leaking water pipes in or around the dwelling?

Expects a single option response (**required**)

☐ Yes [1]

☐ No [2]

Section 7. Indoor environmental conditions

7.1 weather_indoors

When the weather is warm, how does the house feel indoors?

Expects a single option response (**required**)

- ☐ Cooler than outside [1]
- ☐ Same as outside [2]
- ☐ Warmer than outside [3]

7.2 weather_hot

During very hot weather, what do you do to cope? (Respondents can choose more than one)

Expects multiple selected options (**required**)

- ☐ Sleep [1]
- ☐ Sit or lie on the floor indoors [2]
- ☐ Sit under a tree or other shaded areas outdoors [3]
- ☐ Use a fan [4]
- ☐ Use an air conditioner [5]
- ☐ Take a cold bath/shower [6]
- ☐ Drink cold water/ beverages [7]
- ☐ Drink hot tea/coffee/ beverages [8]
- ☐ Remove clothing [9]
- ☐ Open windows [10]
- ☐ Open doors [11]
- ☐ Open curtains/ blinds [12]
- ☐ Switch off lights [13]
- ☐ Do nothing [14]
- ☐ Swim [15]
- ☐ Wet your clothing [16]
- ☐ Other [17]

Prerequisites
Skip when ***weather_hot (7.2)*** **Excludes 'Other [17]'**

7.3 weather_hot_other

You chose other, please specify what you do during very hot weather

Expects a single line text response (**required**)

7.4 weather_hot_children

During hot weather, what do you do to help your children cope? (Respondents can choose more than one)

Expects multiple selected options (**required**)

- ☐ Encourage child to take a nap [1]
- ☐ Sit or lie on the floor indoors [2]
- ☐ Sit under a tree or other shaded areas [3]
- ☐ Use a fan [4]
- ☐ Use an air conditioner [5]
- ☐ Take a cold bath / shower [6]
- ☐ Drink cold water/ beverages [7]
- ☐ Drink hot tea / coffee [8]
- ☐ Remove clothing [9]
- ☐ Open windows [10]
- ☐ Open doors [11]
- ☐ Open curtains/ blinds [12]
- ☐ Switch off lights [13]
- ☐ Do nothing [14]
- ☐ Swim [15]
- ☐ Wet your clothing [16]
- ☐ Other [17]

Prerequisites
Skip when *weather_hot_children (7.4)* Excludes 'Other [17]'

7.5 weather_hot_children_other

You chose other, please specify what you do to help your children cope

Expects a single line text response (**required**)

7.6 weather_cold

During very cold weather, do you use any of the following systems to heat your home?

Expects multiple selected options (**required**)

- ☐ Wood or coal stove [1]
- ☐ Imbhawula [2]
- ☐ Fireplace [3]
- ☐ Gas [4]
- ☐ Electric heater (oil, bar or asbestos) [5]
- ☐ Paraffin heater [6]

Prerequisites
Skip when *weather_cold (7.6)* Excludes 'Wood or coal stove [1]'

7.7 often_wood

How often do you use the Wood / coal stove?

Expects a single option response (**required**)

- ☐ About everyday [1]
- ☐ 2-3 times a week [2]
- ☐ 2-3 times a month [3]
- ☐ Seldom [4]
- ☐ Never [5]

Prerequisites
Skip when *weather_cold (7.6)* Excludes 'Imbhawula [2]'

7.8 often_Imbhawula

How often do you use the Imbhawula?

Expects a single option response (**required**)

- ☐ About everyday [1]
- ☐ 2-3 times a week [2]
- ☐ 2-3 times a month [3]
- ☐ Seldom [4]
- ☐ Never [5]

Prerequisites
Skip when *weather_cold (7.6)* Excludes 'Fireplace [3]'

7.9 often_Fireplace

How often do you use the Fireplace?

Expects a single option response (**required**)

- ☐ About everyday [1]
- ☐ 2-3 times a week [2]
- ☐ 2-3 times a month [3]
- ☐ Seldom [4]
- ☐ Never [5]

Prerequisites
Skip when *weather_cold (7.6)* Excludes 'Gas [4]'

7.10 often_Gas

How often do you use the Gas?

Expects a single option response (**required**)

- ☐ About everyday [1]
- ☐ 2-3 times a week [2]
- ☐ 2-3 times a month [3]
- ☐ Seldom [4]
- ☐ Never [5]

Prerequisites
Skip when *weather_cold (7.6)* Excludes 'Electric heater (oil, bar or asbestos) [5]'

7.11 often_Electric heater

How often do you use the Electric heater?

Expects a single option response (**required**)

- ☐ About everyday [1]
- ☐ 2-3 times a week [2]
- ☐ 2-3 times a month [3]
- ☐ Seldom [4]
- ☐ Never [5]

Prerequisites
Skip when *weather_cold (7.6)* Excludes 'Paraffin heater [6]'

7.12 often_paraffin heater

How often do you use the paraffin heater?

Expects a single option response (**required**)

- ☐ About everyday [1]
- ☐ 2-3 times a week [2]
- ☐ 2-3 times a month [3]
- ☐ Seldom [4]
- ☐ Never [5]

7.13 cooking_daily

Do you do your daily cooking in the

Expects a single option response (**required**)

- ☐ Kitchen (part of the house) [1]
- ☐ Kitchen (separated from the house) [2]
- ☐ Segotlo [3]

7.14 cooking_fuel

What fuel do you mainly use for cooking?

Expects a single option response (**required**)

- ☐ Electricity [1]
- ☐ Paraffin [2]
- ☐ Gas [3]
- ☐ Wood [4]
- ☐ Coal [5]
- ☐ Crop waste / cow dung [6]
- ☐ Other [7]

Prerequisites
Skip when *cooking_fuel (7.14)* Not Equal 'Other [7]'

7.15 cooking_daily_other

You chose other, please specify what fuel you use for cooking

Expects a single line text response (**required**)

7.16 together_family

Does the family sit in the same room around the stove or cooking fire - when cooking takes place?

Expects a single option response (**required**)

- ☐ Yes [1]
- ☐ No [2]

7.17 together_children

Are children and babies in the same room- around the stove or cooking fire - when cooking is taking place?

Expects a single option response (**required**)

- ☐ Yes [1]
- ☐ No [2]

7.18 fire_burn

If you use an open fire, for how many hours are the fires kept burning?

Expects a numeric response (**required**)

7.19 fire_per day

How many times per day is a fire made?

Expects a numeric response (**required**)

7.20 fire_time

Please specify when fires are made

Expects multiple selected options (**required**)

- ☐ Morning [1]
- ☐ Midday [2]
- ☐ Evening [3]

Section 8. Personel and domestic hygiene

8.1 fruit_buy

Where do you mainly get your fruit and vegetables from?

Expects multiple selected options (**required**)

- ☐ Shop [1]
- ☐ Roadside vendor [2]
- ☐ Own garden [3]

8.2 fruit_wash

Do you and members of your household wash raw fruit and vegetables before eating it?

Expects a single option response (**required**)

- ☐ Always [1]
- ☐ Often [2]
- ☐ Sometimes [3]
- ☐ Never [4]

8.3 food_store

If you store food, do you store it in a sealed container?

Expects a single option response (**required**)

- ☐ Yes [1]
- ☐ No [2]

8.4 food_store_container

When you need to store cooked/perishable food, do you store the food in a

Expects a single option response (**required**)

- ☐ Fridge [1]
- ☐ Food cupboard [2]
- ☐ Other [3]

8.5 clean_toilet

How often do you clean your toilet?

Expects a single option response (**required**)

- ☐ Daily [1]
- ☐ Weekly [2]
- ☐ Seldom [3]

8.6 food_soil

Do you or any of your household members eat soil?

Expects a single option response (**required**)

- ☐ Yes [1]
- ☐ No [2]

Prerequisites
Skip when *food_soil (8.6)* **Not Equal 'Yes [1]'**

8.7 food_soil_person

Please specify by person code, who. (P1, P2 ect)

Expects multiple selected options (**required**)

- ☐ P1 [1]
- ☐ P2 [2]
- ☐ P3 [3]
- ☐ P4 [4]
- ☐ P5 [5]
- ☐ P6 [6]
- ☐ P7 [7]
- ☐ P8 [8]
- ☐ P9 [9]
- ☐ P10 [10]

8.8 food_nonfood

Does anyone in household eat other non-food items?

Expects a single option response (**required**)

- ☐ Yes [1]
- ☐ No [2]

Branches

If response **Not Equal 'Yes [1]'** then skip to ***nonfood_nails (8.14)***

8.9 food_nonfood_specify

If yes, please specify- type of non-food item

(**required**)

8.10 nonfood_age

What age is the person? (in years)

Expects a numeric response (**required**)

8.11 nonfood_sex

Is the person male or female?

Expects a single option response (**required**)

- ☐ male [1]
- ☐ female [2]

8.12 nonfood_duration

For how long has this person being eating this?

(**required**)

8.13 nonfood_pregnancy

Was it done during pregnancy?

Expects a single option response (**required**)

- ☐ Yes [1]
- ☐ No [2]

8.14 nonfood_nails

Does anyone in this household especially children bite their nails or suck fingers?

Expects a single option response (**required**)

- ☐ Yes [1]
- ☐ No [2]

Repeat this section for value of ***people_main (2.8)***

Section 9. Health status

9.1 health_symptoms

Has REPEAT IDX experienced the following symptoms related to high temperature in the past month?

Expects multiple selected options (**required**)

- ☐ Diarrhoea [1]
- ☐ Dizziness [2]
- ☐ Headaches [3]
- ☐ Nausea [4]
- ☐ Vomiting [5]
- ☐ Rapid breathing [6]
- ☐ Weakness in the body [7]
- ☐ Fatigue [8]
- ☐ Fainting [9]
- ☐ Fever [10]
- ☐ Hot dry skin [11]
- ☐ Lack of sweating [12]
- ☐ Profuse sweating [13]
- ☐ Dark coloured urine [14]
- ☐ None of the above [15]

Prerequisites
Skip when *health_symptoms (9.1)* Excludes 'Diarrhoea [1]'

9.2 treatment_Diarrhoea

Where did you go for treatment for Diarrhoea?

Expects multiple selected options (**required**)

- ☐ Did not seek medical treatment at all [1]
- ☐ Clinic [2]
- ☐ Doctor [3]
- ☐ Traditional healer [4]
- ☐ Self-medicated [5]
- ☐ Other [6]

Prerequisites
Skip when *health_symptoms (9.1)* Excludes 'Dizziness [2]'

9.3 treatment_Dizziness

Where did you go for treatment for Dizziness?

Expects multiple selected options (**required**)

- ☐ Did not seek medical treatment at all [1]
- ☐ Clinic [2]
- ☐ Doctor [3]
- ☐ Traditional healer [4]
- ☐ Self-medicated [5]
- ☐ Other [6]

Prerequisites
Skip when *health_symptoms (9.1)* Excludes 'Headaches [3]'

9.4 treatment_Headaches

Where did you go for treatment for Headaches?

Expects multiple selected options (**required**)

- ☐ Did not seek medical treatment at all [1]
- ☐ Clinic [2]
- ☐ Doctor [3]
- ☐ Traditional healer [4]
- ☐ Self-medicated [5]
- ☐ Other [6]

Prerequisites
Skip when *health_symptoms (9.1)* Excludes 'Nausea [4]'

9.5 treatment_Nausea

Where did you go for treatment for Nausea?

Expects multiple selected options (**required**)

- ☐ Did not seek medical treatment at all [1]
- ☐ Clinic [2]
- ☐ Doctor [3]
- ☐ Traditional healer [4]
- ☐ Self-medicated [5]
- ☐ Other [6]

Prerequisites
Skip when *health_symptoms (9.1)* Excludes 'Vomiting [5]'

9.6 treatment_Vomiting

Where did you go for treatment for Vomiting?

Expects multiple selected options (**required**)

- ☐ Did not seek medical treatment at all [1]
- ☐ Clinic [2]
- ☐ Doctor [3]
- ☐ Traditional healer [4]
- ☐ Self-medicated [5]
- ☐ Other [6]

Prerequisites
Skip when *health_symptoms (9.1)* Excludes 'Rapid breathing [6]'

9.7 treatment_Rapid breathing

Where did you go for treatment for Rapid breathing?

Expects multiple selected options (**required**)

- ☐ Did not seek medical treatment at all [1]
- ☐ Clinic [2]
- ☐ Doctor [3]
- ☐ Traditional healer [4]
- ☐ Self-medicated [5]
- ☐ Other [6]

Prerequisites
Skip when *health_symptoms (9.1)* Excludes 'Weakness in the body [7]'

9.8 treatment_Weakness in the body

Where did you go for treatment for Weakness in the body?

Expects multiple selected options (**required**)

- ☐ Did not seek medical treatment at all [1]
- ☐ Clinic [2]
- ☐ Doctor [3]
- ☐ Traditional healer [4]
- ☐ Self-medicated [5]
- ☐ Other [6]

Prerequisites
Skip when *health_symptoms (9.1)* Excludes 'Fatigue [8]'

9.9 treatment_Fatigue

Where did you go for treatment for Fatigue?

Expects multiple selected options (**required**)

- ☐ Did not seek medical treatment at all [1]
- ☐ Clinic [2]
- ☐ Doctor [3]
- ☐ Traditional healer [4]
- ☐ Self-medicated [5]
- ☐ Other [6]

Prerequisites
Skip when *health_symptoms (9.1)* Excludes 'Fatigue [8]'

9.10 treatment_Fainting

Where did you go for treatment for Fainting?

Expects multiple selected options (**required**)

- ☐ Did not seek medical treatment at all [1]
- ☐ Clinic [2]
- ☐ Doctor [3]
- ☐ Traditional healer [4]
- ☐ Self-medicated [5]
- ☐ Other [6]

Prerequisites
Skip when *health_symptoms (9.1)* Excludes 'Fever [10]'

9.11 treatment_Fever

Where did you go for treatment for Fever?

Expects multiple selected options (**required**)

- ☐ Did not seek medical treatment at all [1]
- ☐ Clinic [2]
- ☐ Doctor [3]
- ☐ Traditional healer [4]
- ☐ Self-medicated [5]
- ☐ Other [6]

Prerequisites
Skip when *health_symptoms (9.1)* Excludes 'Hot dry skin [11]'

9.12 treatment_Hot dry skin

Where did you go for treatment for Hot dry skin?

Expects multiple selected options (**required**)

- ☐ Did not seek medical treatment at all [1]
- ☐ Clinic [2]
- ☐ Doctor [3]
- ☐ Traditional healer [4]
- ☐ Self-medicated [5]
- ☐ Other [6]

Prerequisites
Skip when *health_symptoms (9.1)* Excludes 'Lack of sweating [12]'

9.13 treatment_Lack of sweating

Where did you go for treatment for Lack of sweating?

Expects multiple selected options (**required**)

- ☐ Did not seek medical treatment at all [1]
- ☐ Clinic [2]
- ☐ Doctor [3]
- ☐ Traditional healer [4]
- ☐ Self-medicated [5]
- ☐ Other [6]

Prerequisites
Skip when *health_symptoms (9.1)* Excludes 'Profuse sweating [13]'

9.14 treatment_Profuse sweating

Where did you go for treatment for Profuse sweating?

Expects multiple selected options (**required**)

- ☐ Did not seek medical treatment at all [1]
- ☐ Clinic [2]
- ☐ Doctor [3]
- ☐ Traditional healer [4]
- ☐ Self-medicated [5]
- ☐ Other [6]

Prerequisites
Skip when *health_symptoms (9.1)* Excludes 'Dark coloured urine [14]'

9.15 treatment_Dark coloured urine

Where did you go for treatment for Dark coloured urine?

Expects multiple selected options (**required**)

- ☐ Did not seek medical treatment at all [1]
- ☐ Clinic [2]
- ☐ Doctor [3]
- ☐ Traditional healer [4]
- ☐ Self-medicated [5]
- ☐ Other [6]

9.16 health_other

Is there another place where you go for treatment?

Expects a single option response (**required**)

☐ Yes [1]

☐ No [2]

Prerequisites
Skip when *health_other (9.16)* Not Equal 'Yes [1]'

9.17 health_other_specify

Please specify where you go for treatment

(**required**)

Repeat this section for value of ***people_main (2.8)***

Section 10. Past medical history

10.1 medical_history

Has REPEAT IDX in the past year been diagnosed with any of the following illnesses?

Expects multiple selected options (**required**)

- ☐ Hypertension EVER [1]
- ☐ Diabetes EVER [2]
- ☐ Heart disease EVER [3]
- ☐ Asthma EVER [4]
- ☐ Chronic Lung disease EVER [5]
- ☐ Pneumonia [6]
- ☐ Tuberculosis [7]
- ☐ High Cholesterol EVER [8]
- ☐ Anaemia [9]
- ☐ HIV (EVER) [10]

10.2 medical_smoke

Does REPEAT IDX use tobacco?

Expects a single option response (**required**)

- ☐ Yes [1]
- ☐ No [2]

Branches

If response **Not Equal 'Yes [1]'** then skip to ***medical_alcohol (10.5)***

10.3 medical_tabacco

What type of tobacco?

Expects multiple selected options (**required**)

- ☐ Cigarettes [1]
- ☐ Pipe [2]
- ☐ Snuff [3]
- ☐ Chewing tobacco [4]
- ☐ Hookah pipe [5]
- ☐ Other [6]

Prerequisites
Skip when ***medical_tabacco (10.3)*** **Excludes 'Other [6]'**

10.4 medical_tabacco_specify

Please specify what type of tabacco

(**required**)

10.5 medical_alcohol

Does REPEAT IDX drink alcohol?

Expects a single option response (**required**)

- ☐ Yes [1]
- ☐ No [2]

Section 11. Medical history 2

11.1 medical_died

Has anyone in this dwelling died in the last 12 months?

Expects a single option response (**required**)

☐ Yes [1]

☐ No [2]

Branches

If response **Not Equal 'Yes [1]'** then skip to ***medical_breastfed (11.5)***

11.2 medical_died_age

If yes, how old was he/she?

Expects a numeric response (**required**)

11.3 medical_died_sex

Please state sex

Expects a single option response (**required**)

☐ Male [1]

☐ Female [2]

11.4 medical_died_cause

What did he/she die of?

(**required**)

11.5 medical_breastfed

How many children are currently being breastfed?

Expects a numeric response (**required**)

Repeat this section for value of ***medical_breastfed (11.5)***

Section 12. Breastfeading

12.1 breastfed_duration

Was REPEAT IDX exclusively breastfed in the first six month of his/her life?

Expects a single option response (**required**)

☐ Yes [1]

☐ No [2]

Section 13. Malaria risk assessment

13.1 malaria_prevention

What is done to prevent a person from getting malaria?

Expects multiple selected options (**required**)

- ☐ Use bed nets [1]
- ☐ Wear protective clothing [2]
- ☐ Use insect repellent [3]
- ☐ Use flying-insect spray indoors [4]
- ☐ Take medicine before and after visiting malaria areas [5]

13.2 malaria_spray

At any time in the past 12 months, have the inside walls of your home been sprayed?

Expects a single option response (**required**)

- ☐ Yes [1]
- ☐ No [2]

Branches

If response **Equals 'No [2]'** then skip to ***mosquitoes_inside (13.5)***

13.3 malaria_spray_wash

Did you wash or clean your walls within 1 month after spraying?

Expects a single option response (**required**)

- ☐ Yes [1]
- ☐ No [2]

13.4 malaria_walls

Since your walls have been sprayed, have you covered the walls with anything such as new paint or plaster?

Expects a single option response (**required**)

- ☐ Yes [1]
- ☐ No [2]

13.5 mosquitoes_inside

What do you use to get rid of mosquitoes that are inside your house?

Expects multiple selected options (**required**)

- ☐ Insecticidal sprays [1]
- ☐ Vaporisation mats [2]
- ☐ Mosquito coils [3]
- ☐ Ceiling fans or air conditioners [4]
- ☐ Nothing [5]

13.6 mosquitoes_nets

Do you use mosquito nets in your home?

Expects a single option response (**required**)

- ☐ Yes [1]
- ☐ No [2]

Branches

If response **Not Equal 'Yes [1]'** then skip to ***insect_repellent (13.9)***

13.7 mosquitoes_nets_condition

If you use nets, what is the condition of the mosquito nets?

Expects a single option response (**required**)

- ☐ no holes [1]
- ☐ 1-4 holes [2]
- ☐ > 5 holes [3]
- ☐ Unused [4]

13.8 mosquitoes_nets_treated

Do you know if the nets that you used have been treated with chemicals to kill mosquitoes?

Expects a single option response (**required**)

- ☐ Yes [1]
- ☐ No [2]
- ☐ Don't know [3]

13.9 insect_repellent

Do you use insect repellent to stop mosquitoes from biting you?

Expects a single option response (**required**)

- ☐ Yes [1]
- ☐ No [2]

13.10 dwelling_water

Is your house within 100m of a marshy area or water body?

Expects a single option response (**required**)

- ☐ Yes [1]
- ☐ No [2]

13.11 dwelling_water_drainage

Does your house have good drainage of rainwater and household water away from the house?

Expects a single option response (**required**)

- ☐ Yes [1]
- ☐ No [2]

13.12 evening_out

Do you or any members of your household often go out at night/in the evening or spend time outside your house in the evening?

Expects a single option response (**required**)

- ☐ Yes [1]
- ☐ No [2]

Section 14. Population movement

14.1 movement_travel

Have you or members of the household travelled to another country or province in the past 6 months?

Expects a single option response (**required**)

☐ Yes [1]

☐ No [2]

Branches

If response **Not Equal 'Yes [1]'** then skip to ***movement_family (14.5)***

14.2 movement_person

Please specify who

Expects a single line text response (**required**)

14.3 movement_country

If yes, which country/province was visited?

Expects a single line text response (**required**)

14.4 movement_malaria

Among those who travelled, did they take any medicine to prevent getting malaria?

Expects a single option response (**required**)

☐ Yes [1]

☐ No [2]

14.5 movement_family

Do you have a friend or family member from another province or country living with you from time to time?

Expects a single option response (**required**)

☐ Yes [1]

☐ No [2]

Prerequisites

Skip when ***movement_family (14.5)*** **Not Equal 'Yes [1]'**

14.6 movement_family_province

If yes, which country/province do they come from?

Expects a single line text response (**required**)

Section 15. End

15.1 end

This is the end of the questionnaire. Thank you very much for your participation.
